# Topography, management, and extreme precipitation influence greenhouse gas emissions in a cool, humid corn silage system

**DOI:** 10.1002/jeq2.70187

**Published:** 2026-04-29

**Authors:** Molly Ratliff, Joshua W. Faulkner, Eric D. Roy, Marie English, Dan Liptzin, Reza K. Afshar, E. Carol Adair

**Affiliations:** ^1^ Rubenstein School of Environment and Natural Resources University of Vermont Burlington Vermont USA; ^2^ Extension Center for Sustainable Agriculture University of Vermont Burlington Vermont USA; ^3^ Department of Agriculture, Landscape & Environment, College of Agriculture and Life Sciences University of Vermont Burlington Vermont USA; ^4^ Gund Institute for Environment University of Vermont Burlington Vermont USA; ^5^ Department of Civil & Environmental Engineering University of Vermont Burlington Vermont USA; ^6^ Soil Health Institute Morrisville North Carolina USA; ^7^ Dairy Research Institute Rosemont Illinois USA

## Abstract

Agricultural soils are sources of nitrous oxide (N_2_O) and, under prolonged saturation, methane (CH_4_)—two potent greenhouse gases (GHGs). Soil management, field topography, and climate all influence GHG emissions, yet their interactions are not well understood. Over 17 months, we evaluated how three distinct management systems—Conventional, a soil health system (Soil Health), and a flocculated manure solid amendment (Flocculated Solids)—interacted with topographically high and low areas to influence N_2_O and CH_4_ emissions in a 21 ha corn (*Zea mays* L.) silage field in western Vermont, during a period of abnormally high precipitation. At 18 plots (3 treatments × 2 topographic positions × 3 replicates), we measured GHG fluxes year‐round alongside soil temperature, moisture, and inorganic nitrogen. Annual N_2_O emissions were 4.4 times greater in Soil Health‐Low plots (74.3 kg N_2_O‐N ha^−1^ year^−1^) than in Flocculated Solids plots, which had the lowest emissions (17.0 kg N_2_O‐N ha^−1^ year^−1^). Annual CH_4_ emissions were greatest in low plots across all treatments, with low plots emitting 2.2 times more CH_4_ than high plots. Boosted regression tree models identified soil moisture, ammonium, CO_2_ flux, and nitrate as the strongest predictors of daily N_2_O fluxes. For CH_4_, inundation duration was the dominant driver, with emissions increasing sharply after >40 days of continuous saturation. Treatment and topography explained <5% of emissions in both models, indicating that their effects are primarily indirect, modifying soil moisture, nitrogen availability, and organic matter inputs that ultimately drive GHG emissions.

## Core Ideas

- Heavy rainfall and poorly drained clay soils often resulted in prolonged soil saturation during this study.
- Soil moisture and inorganic nitrogen concentrations were key drivers of greenhouse gas emissions.
- Soil health‐low plots exhibited the greatest nitrous oxide emissions.
- Methane emissions were greatest in the low‐lying areas of the field across all management systems.

## Plain Language Summary

Greenhouse gases are naturally released from soil. However, the rate and magnitude of these emissions depend on how agricultural soils are managed, where they are within a field, and weather conditions. We studied nitrous oxide and methane emissions in a Vermont cornfield managed under three different systems across high and low landscape positions during an unusually wet growing season. Emissions were greatest in the wetter, low‐lying areas of the field where liquid manure was injected into the soil and lowest where a drier manure product was applied at a lower nitrogen rate. Soil moisture and available nutrients were the strongest factors controlling emissions, meaning that practices that increase nitrogen retention or soil wetness may also increase gaseous losses. These findings highlight the need for field‐specific management that accounts for environmental conditions and landscape position, especially as extreme precipitation becomes more common with climate change.

**Abbreviations**
BRT: boosted regression tree
GHG: greenhouse gas

## 1 INTRODUCTION

Agriculture now occupies nearly half of all habitable land (United States Department of Agriculture Economic Research Service, 2025). While food production sustains a growing global population, it also contributes substantially to greenhouse gas (GHG) emissions. Agricultural soils are a leading source of nitrous oxide (N_2_O) emissions, a GHG that is almost 300 times more powerful at trapping heat than carbon dioxide (CO_2_) (Intergovernmental Panel on Climate Change [IPCC], 2013; Syakila & Kroeze, 2011). Similarly, agricultural activities contribute substantially to anthropogenic methane (CH_4_) emissions, another powerful GHG (IPCC, 2014; Smith et al., 2021). Identifying the conditions and soil management strategies that maintain agricultural productivity while minimizing GHG emissions is a critical focus of climate change mitigation research.

Soil health practices are widely promoted as climate‐smart strategies that increase soil organic matter, enhance soil structure, and reduce nutrient losses while maintaining yields in feed production systems (Hatano et al., 2024; Lehmann et al., 2020; Paustian et al., 2016). However, research shows that building soil health can introduce tradeoffs, such as increasing N_2_O emissions (Dittmer et al., 2020; van Kessel et al., 2013). Cover cropping and reduced tillage improve soil health by retaining more carbon (C) and nitrogen (N) in the soil, but this increased substrate availability can enhance microbial activity that produces N_2_O (Grave et al., 2018; Pugesgaard et al., 2017; Regina & Alakukku, 2010; C. Wang et al., 2021). Similarly, manure injection, while beneficial for reducing ammonia volatilization and improving nutrient retention, can elevate N_2_O emissions relative to surface broadcasting by creating localized anaerobic zones rich in both C and N—ideal conditions for denitrification (Agnew et al., 2010; Butterbach‐Bahl et al., 2013; Dittmer et al., 2020; Duncan et al., 2017; Montes et al., 2013; Ponce de León et al., 2021; Sherman et al., 2022). While individual practices have been widely studied, the combined effects of stacked management strategies on field‐scale GHG emissions remain poorly understood—despite their potential to address multiple environmental outcomes through different mechanisms (Cloutier et al., 2025; O'Brien et al., 2022; White et al., 2023).

Because management practices will interact with environmental conditions to enhance or mitigate GHG emissions, it is critical to understand the role of field topography in modulating N_2_O and CH_4_ fluxes (Chapuis‐Lardy et al., 2007; Oertel et al., 2016). Even in uniformly managed fields, topographic variation causes differences in sediment deposition, water flow patterns, and decomposition rates, which result in field‐scale variation in soil properties and biogeochemical processes (M. Zhang, Fu, et al., 2024; Shukla et al., 2023; Z. Zhang, Eddy, et al., 2024). Low areas, which accumulate more water, organic matter, and nutrients, are often identified as hotspots of denitrification (Ball, 2013; Murphy et al., 2009; Vilain et al., 2010; M. Wang et al., 2023). Conversely, relatively high areas often retain less moisture, resulting in more aerobic conditions and lower N_2_O emissions (Negassa et al., 2015; Suriyavirun et al., 2019). However, this topographic hotspot effect may be seasonally dependent, with low areas emitting the most N_2_O early in the growing season when excess N from fertilization may be present in the soil and declining later in the growing season as plant uptake reduces nitrate availability (Krichels et al., 2019; Molodovskaya et al., 2012; Saha et al., 2017). Beyond these seasonal patterns, GHG fluxes can also vary sharply over shorter timescales as rainfall, thawing, or drying–rewetting events further alter soil oxygen and substrate dynamics, producing transient emissions that increase background rates (Elberling et al., 2023; Groffman et al., 2009; Guo et al., 2014; Li et al., 2021). Despite this understanding, research that assesses the extent to which topography influences GHG emissions across different soil types, seasons, and upland cropping systems is still limited. Furthermore, the role of topography in determining GHG emissions is especially relevant in the undulating northeastern United States, where the frequency and intensity of extreme precipitation have increased in recent decades and are projected to rise further with climate change (Hicke et al., 2022; Walthall et al., 2012).

Methane (CH_4_) dynamics in upland agricultural soils remain largely understudied, as upland soils often act as net CH_4_ sinks, taking up more CH_4_ than they emit (Hansen et al., 2024; Maljanen et al., 2006). However, with increasing extreme precipitation events, methane production from upland soils may become more common. Following extended periods of anoxia, often 30 days or longer, CH_4_ pulses have been measured (Brewer et al., 2018; Sánchez‐Rodríguez et al., 2019; Scott et al., 2022). Studies in natural systems have also demonstrated that topographically low, carbon‐rich areas produce higher CH_4_ fluxes than high topographic areas, a pattern that may extend to agricultural fields with persistent wetness (Bubier et al., 1993; Perryman et al., 2022). Additionally, high methane fluxes may be observed in anaerobic agricultural soils because high ammonium (NH_4_^+^) concentrations can competitively inhibit CH_4_ oxidation, due to similarities in chemical structure (Aronson & Helliker, 2010; Chan & Parkin, 2001; Maljanen et al., 2006). These observations suggest that CH_4_ emissions, like N_2_O, are shaped by interacting biological and environmental controls that vary across space and time.

Emerging research highlights the complex and variable nature of N_2_O and CH_4_ fluxes across agricultural landscapes, where management practices and environmental conditions modulate nitrogen availability, soil organic matter, and oxygen status, all of which vary across topography. Yet, most studies occur in uniform experimental plots, overlooking these topographic influences. As a result, little is known about how topographic variability modulates the effectiveness of management practices in reducing GHG emissions from upland soils. The objective of this study was to evaluate how three different soil management systems—Conventional, a soil health management system (Soil Health), and a flocculated manure solid amendment (Flocculated Solids)—interact with topographic position (high vs. low) to regulate N_2_O and CH_4_ emissions in a corn (*Zea mays* L.) silage field. These treatments represent distinct field‐scale systems, including conventional and conservation‐based approaches, along with a novel manure amendment designed to enhance nutrient use efficiency. Additionally, by leveraging a dataset collected during a period of record‐breaking rainfall, this field research offers a unique opportunity to evaluate GHG fluxes under real‐world climate extremes—a key component for improving GHG budgets and developing effective mitigation strategies in the face of climate change. We hypothesized that N_2_O and CH_4_ emissions would be greatest in the soil health treatment due to increased substrate availability and soil moisture retention resulting from the combined effects of cover cropping, no‐till, and manure injection. We further hypothesized that topographically low areas would also exhibit higher CH_4_ and N_2_O emissions than high areas, also due to prolonged saturation and greater carbon and nitrogen availability.

**Core Ideas**

- Heavy rainfall and poorly drained clay soils often resulted in prolonged soil saturation during this study.
- Soil moisture and inorganic nitrogen concentrations were key drivers of greenhouse gas emissions.
- Soil health‐low plots exhibited the greatest nitrous oxide emissions.
- Methane emissions were greatest in the low‐lying areas of the field across all management systems.

## 2 MATERIALS AND METHODS

### 2.1 Site description

This study was conducted from July 2023 to November 2024 on a dairy farm in Bridport, VT. The study site was a 21‐ha corn silage field rotated with mixed‐species grass hay every 4–5 years. The field was continuously planted with corn from spring of 2021 through the study period. Prior to the start of the study, existing management included no‐till planting and fall liquid dairy manure injection. Artificial subsurface pattern drainage (i.e., tile drainage) was installed in the field approximately 10 years prior to this study. The field site had a mean annual temperature of 9.5°C in 2023 and 9.7°C in 2024. Total annual precipitation was 1333.7 mm in 2023 and 1061.2 mm in 2024, both exceeding the 30‐year average of 1001.7 mm (Figure S1). During the growing season (May–September), mean daily temperatures ranged from 11.0°C to 27.7°C, while mean daily precipitation ranged from 0 to 91.4 mm. Soils at the site are classified as poorly drained Covington and Panton silty clays and moderately well‐drained Vergennes clays. Prior to initiating treatments, soil samples across the entire field indicated an average bulk density of 1.1 g cm^−3^; pH of 6.1; 3.0% total C; and 0.3% total N (June 2023, 0–15 cm), with corresponding mean (± standard deviation [SD]) values by topographic position summarized in Table S1.

The field was divided into three large treatment blocks, each representing different management strategies (Figure S2). While the treatments were not conventionally replicated due to field‐scale constraints of the study, each treatment block contained six spatially distributed plots (20 m radius) designated for repeated GHG measurements and soil sampling. Within each treatment, three plots were located in topographically high areas and three in topographically low areas (3 treatments × 2 topographic positions × 3 replicates). Plot locations were selected based on elevation data derived from high‐resolution satellite imagery and field verification. The three treatments represented contrasting approaches to manure and soil management, designed to reflect both typical and emerging practices in the region. Descriptions of each treatment are as follows:

1. *Conventional*: Conventional practices including fall broadcast of liquid dairy manure with immediate chisel plow incorporation and secondary springtime tillage.
2. *Soil Health*: Stacked conservation practices including no‐till planting, winter wheat (*Triticum aestivum* L.) cover crop, and fall liquid dairy manure injection.
3. *Flocculated Solids*: Identical management to Soil Health, with the exception that liquid dairy manure was replaced by flocculated manure solids recovered from a dissolved air flotation process that separates fine particles from dairy manure digestate (Porterfield et al., 2020). The Flocculated Solids were surface‐applied.

Each treatment was implemented across a large portion of the field: approximately 4.2 ha for Conventional, 7.4 ha for Soil Health, and 2.5 ha for Flocculated Solids. Full management treatments were initiated on October 5, 2023. All manure treatments were applied on a phosphorus (P) basis to prevent excessive P loading to the field. Because the Flocculated Solids have a much higher P:N ratio than liquid manure, applying them at rates sufficient to match N availability would greatly exceed crop P requirements. In Vermont, nutrient management guidelines emphasize avoiding excessive P application due to risks of eutrophication and runoff (University of Vermont Extension, 2018). Vermont certified farms are required to adopt field‐by‐field nutrient management plans under NRCS 590, which prioritize matching P to crop removal to protect water quality, constraining the application rate of P‐rich Flocculated Solids in this field study (Natural Resources Conservation Service, 2023; Vermont Agency of Agriculture, Food & Markets, n.d.). This approach resulted in higher manure‐derived N applied to the treatments receiving liquid manure, and lower manure‐derived N applied to the Flocculated Solids treatment. Manure rates and nutrient contents from each treatment block are detailed in Table 1. In addition to manure application, all treatments received a uniform sidedress application of urea (∼85 kg N ha^−1^ of 38‐0‐0) in late June of each growing season (Table S2). Treatments were planted and harvested on the same schedule, but differed in their use of tillage, cover cropping, and manure type or application method (Table S2). Corn silage yield was measured at harvest using a yield monitor and summarized at the plot level.

**TABLE 1:** Manure application rates, physical characteristics, and nutrient contents.

Manure characteristic	Oct. 5, 2023	Sept. 24, 2024
^a^ **Liquid dairy manure**		
Application rate (L ha^−1^)	140234.3	140234.3
Dry matter (%)	1.7	2.5
Total N (kg ha^−1^)	289.8	289.8
NH4‐N (kg ha^−1^)	152.9	107.5
Organic N (kg ha^−1^)	136.9	184.0
Total P (kg ha^−1^)	18.3	26.0
**Flocculated solids**		
Application rate (kg ha^−1^)	6725.1	6725.1
Dry matter (%)	21.9	25.7
Total N (kg ha^−1^)	63.9	77.7
NH4‐N (kg ha^−1^)	7.7	7.4
Organic N (kg ha^−1^)	56.2	70.6
Total P (kg ha^−1^)	23.2	26.5

^a^ Manure properties averaged across the liquid dairy manure applied to Conventional and Soil Health plots.

### 2.2 Gas flux measurements

Soil N_2_O, CO_2_, and CH_4_ emissions were measured approximately weekly during the growing season and biweekly in the early spring and late fall. Following manure application, sampling increased to every other day for 14 days. During the winter, sampling occurred monthly. Treatment blocks were sampled in a random order and flux measurements were conducted between ∼8:00 a.m. and 1:00 p.m. In each plot, two beveled‐edge polyvinylchloride collars (20 cm diameter, 12.5 cm height, and 0.7 cm thick) were installed: one within the corn rows (I) and one between rows (O), approximately 1 m apart (36 total collars). Collars were removed only before management activities, then reinstalled in new locations within the same plot, maintaining consistent in‐/out‐of‐row placement. Collars were generally reinstalled at least 24 h before sampling to minimize disturbance‐related fluxes. Prior to each gas measurement, collar height above the soil surface was recorded to calculate headspace volume for flux estimation.

Fluxes were measured using LI‐COR Trace Gas Analyzers (LI‐7810 N_2_O/H_2_O, LI‐7820 CO_2_/CH_4_/H_2_O) and Smart Chamber (8200‐01S) via Optical Feedback—Enhanced Absorption Spectroscopy (LI‐COR). Each measurement was 120 s, including a deadband period to adjust for pressure disturbance to the system upon chamber closure. Daily GHG data from the LI‐COR analyzers were merged and preprocessed in SoilFluxPro 5.3.0 (SFP). Gas fluxes were calculated in the SFP software using the following equation:

$$F_{c}=\frac{10VP_{0}\left(1-\frac{W_{0}}{1000}\right)}{RS\left(T_{0}+273.15\right)}\times \frac{\partial C\prime }{\partial t}$$

where *F_c_* is the flux rate in µmol m^−2^ s^−1^ or nmol m^−2^ s^−1^, *V* is total volume of the system (cm^3^), *P_0_* is pressure at time zero (kPa), *R* is the gas constant (8.314 Pa m^3^ K^−1^), *S* is surface area of the soil (cm^2^), *T*_0_ is the temperature at time zero (°C), and 𝜕*C′/*𝜕*t* is the rate of change of the dilution‐corrected mole fraction of gas with respect to time (LI‐COR Biosciences, n.d.). Soil moisture and temperature were also captured during each gas measurement.

To approximate annual N_2_O and CH_4_ emissions, cumulative fluxes were calculated for the first full year of treatment data (October 6, 2023–October 6, 2024). Missing flux values resulting from instrument error or plot inundation (GHG sampling collars submerged under standing water) were imputed using group‐level means based on date, treatment, and topographic position to complete the dataset. When these grouping factors were not applicable, data were averaged across date and topography. Because peak flux events often coincided with periods of extreme wetness, this approach likely resulted in a modest underestimation of cumulative emissions. However, the same imputation procedure was applied uniformly across all experimental groups, so any underestimation is expected to be consistent across treatments. Following imputation, cumulative emissions were estimated by integrating the area under the daily flux curve over time using the pracma package in R (Borchers, 2022; RStudio Team, 2022).

### 2.3 Soil sampling and nitrogen availability

A soil sample (0‐ to 15‐cm depth, 2 cm diameter) was taken at each plot during gas sampling for inorganic nitrogen analyses. Samples were homogenized and placed on ice. Within 24 h of sampling, 5‐g field wet subsamples were extracted with 2 M potassium chloride (KCl). Extracts were frozen until analysis for nitrate (NO_3_^−^) and ammonium (NH_4_^+^) using colorimetric analysis (BioTek Synergy HTX, BioTek Instruments, Inc.; Forester, 1995). Gravimetric soil moisture was determined by oven drying 5‐g field wet subsamples at 60°C to a constant weight. Soil samples were not taken during the winter months.

### 2.4 Data analysis

Differences in daily N_2_O fluxes and CH_4_ fluxes between topographic position, management, and their interaction were analyzed for the full study period using linear mixed effects models with chamber as a random effect (lme4 package; Bates et al., 2015; RStudio Team, 2022). Both models included variance structures for management and topography, and an autoregressive correlation structure to account for temporal correlation. To evaluate whether inclusion of pretreatment observations influenced model outcomes, these analyses were repeated using only post‐treatment data (October 6, 2023, onward). Differences in cumulative emissions between topographic position and management treatment were also analyzed using linear models. All GHG data, except cumulative CH_4_ emissions, were log‐transformed to meet normality assumptions. Untransformed data were used for figures and ease of interpretation. Tukey honestly significant difference (HSD) post hoc tests were used to show cumulative emissions differences.

While linear mixed effects models were useful for testing treatment‐level effects, significance alone did not fully capture the complex and interacting drivers of GHG emissions in this system. To better understand the relative influence of environmental and management variables, we used boosted regression tree (BRT) models to identify key predictors of daily N_2_O and CH_4_ fluxes across the full study period (gbm and caret packages; Greenwell et al., 2020; Kuhn, 2022; RStudio Team, 2022). Predictor variables included in the N_2_O BRT model were soil moisture, soil NH_4_^+^, soil NO_3_^−^, soil temperature, CO_2_ flux (as a proxy for readily available carbon), days post‐manure application, management treatment, topographic position, and corn row position. The CH_4_ model also included inundation duration as a predictor and did not include days post‐manure. Repeated 10‐fold cross‐validation was used in conjunction with a tuning grid to identify optimal model hyperparameters that minimized the root mean squared error in both models. The tuning grid used a range of values for the following parameters: number of trees (n.trees), interaction depth (interaction.depth), learning rate (shrinkage), and minimum number of observations per terminal node (n.minobsinnode). The optimal parameters were then used to rebuild the final model that determined the relative influence of each predictor on N_2_O and CH_4_ fluxes. Partial dependence plots were used to visualize nonlinear relationships of each predictor variable on N_2_O and CH_4_ fluxes. To explore interactions between predictors, bivariate dependence plots were generated for predictor pairs with a Friedman's H‐statistic >0.1, illustrating how the combined influence of two variables affected emissions (Elith et al., 2008; Greenwell et al., 2020; RStudio Team, 2022).

To further interpret the low relative influence of management and topography in the BRT models, we conducted additional linear mixed effects analyses on daily CO_2_ flux, soil moisture, soil NH_4_^+^, and soil NO_3_^−^ concentrations—key environmental variables used as predictors in the BRT models. These models included the same fixed and random effects structure as the GHG flux models, allowing us to test whether these variables differed significantly by treatment, topography, or their interaction. In addition, corn silage yield and N_2_O emissions intensity (cumulative N_2_O divided by yield) were analyzed using two‐way analysis of variances (ANOVAs) with management, topography, and their interaction as fixed effects.

## 3 RESULTS AND DISCUSSION

### 3.1 Environmental drivers of nitrous oxide emissions

Relationships among environmental and management variables showed nonlinear and interdependent patterns (Figure S3), indicating that simple linear approaches could not adequately capture the factors driving GHG emissions. To capture these complex and threshold‐based relationships, we used BRT models to evaluate the relative influence of each variable on daily N_2_O emissions. The most influential predictors (relative influence > 10%; Elith et al., 2008) were soil moisture, soil NH_4_^+^, CO_2_ flux (as a proxy for labile carbon), and soil NO_3_^−^ (Table 2). In contrast, topography, management, and collar position together contributed ˂5% to N_2_O emissions (Table 2), suggesting that treatment and topographic effects on emissions are primarily indirect, mediated through their influence on soil moisture and nitrogen and carbon dynamics (Brickman et al., 2024; Gu et al., 2011).

**TABLE 2:** Hyperparameters, relative influence of predictor variables, and error from daily N_2_O and CH_4_ boosted regression tree (BRT) models.

	N_2_O model	CH_4_ model
	Hyperparameters	
n.trees	1500	500
interaction.depth	3	3
shrinkage	0.01	0.001
n.minobsinnode	5	10
	**Relative influence**	
Inundation duration	–	40.0
Soil moisture	26.7	1.9
NH_4_	20.0	25.0
CO_2_	19.7	3.2
NO_3_	12.6	30.2
Days post‐manure application	9.5	–
Soil temperature	6.9	1.6
Management	4.2	1.1
Row	0.3	–
Topo	0.2	0.01
	**Root mean squared error**	
CV	325.7	101.7
Training	217.5	143.9

*Note*: Topo, topography, Row, in‐/out‐of‐row collar position.

Abbreviation: CV, coefficient of variance.

**FIGURE 1:**
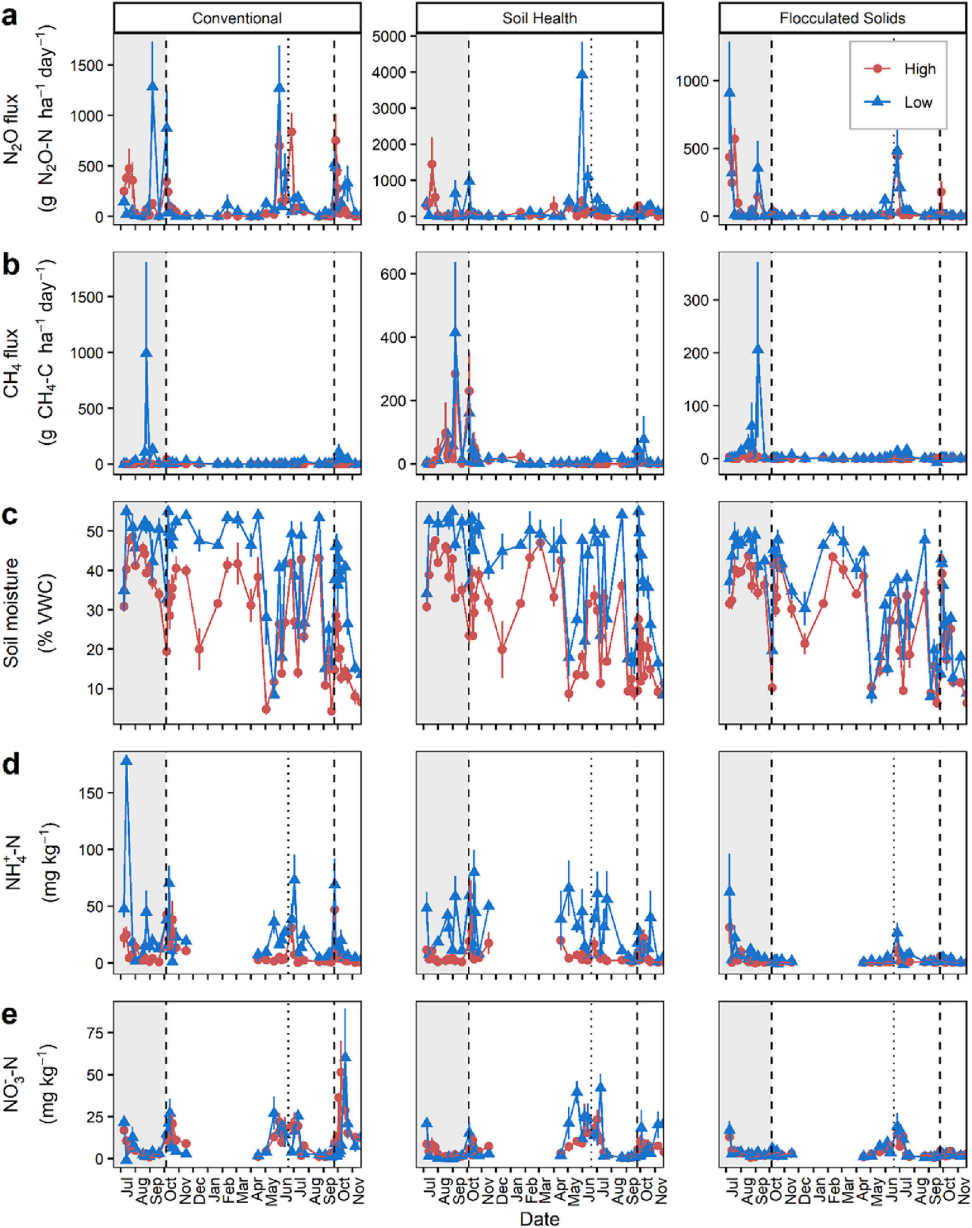
Mean daily N_2_O flux, CH_4_ flux, soil moisture, NH_4_^+^‐N, and NO_3_^−^‐N across management (vertical panels) and topography (color and shape). Vertical dashed lines represent manure application on October 5, 2023 and September 24, 2024. The vertical dotted line represents sidedress application on June 19, 2024. Gray shading indicates the pre‐treatment period. Data from July 2023 to November 2024. Note that *y*‐axis scales differ between treatment panels for N_2_O and CH_4_ fluxes. Soil samples for inorganic nitrogen were not taken during the winter months. VWC, volumetric water content.

Soil moisture alone contributed over 25% to N_2_O emissions (Table 2). Our dataset captured a broad range of soil moisture values (Figure 1c), with predicted N_2_O emissions increasing modestly up to approximately 45% volumetric water content (VWC), then doubling between 45% and 55% and plateauing at higher moisture levels (Figure S4a). This pattern reflects increases in denitrification‐derived N_2_O emission when soils approach saturation and decreases in N_2_O emissions under extreme wetness as complete denitrification or other redox processes take over (Gu et al., 2011; Liu et al., 2022; Pärn et al., 2018; Schaufler et al., 2010; Zheng et al., 2000). However, 45%–55% VWC did not always result in high observed N_2_O fluxes (Figure 1), indicating interacting drivers (Brickman et al., 2024).

**FIGURE 2:**
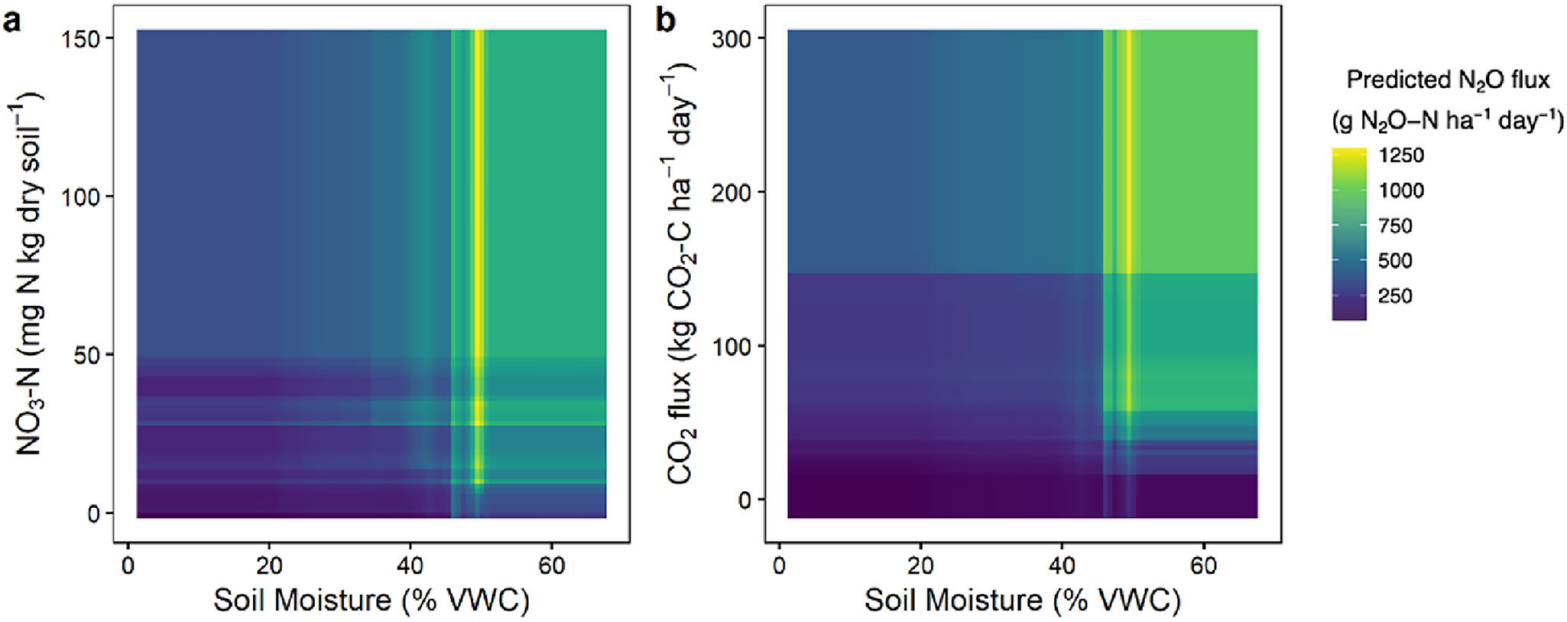
Bivariate dependence plots showing two‐way interactions between (a) soil moisture and soil nitrate concentrations and (b) soil moisture and CO_2_ flux. Lighter areas indicate larger predicted N_2_O fluxes. VWC, volumetric water content.

Soil moisture is the top driver of N_2_O emissions, but only when ample soil NO_3_^−^ and dissolved organic carbon are present, such as in low spots of a field (Pärn et al., 2018; Z. Zhang, Eddy, et al., 2024). Soil inorganic N concentrations were generally greater in low topographic positions through the study period (Figure 1d,e), indicating that these areas accumulated larger pools of available nitrogen. The BRT model predicted higher N_2_O emissions when soil NO_3_^−^ levels were >50 mg N kg dry soil^−1^ and when soil moisture was >45% VWC (Figure 2a). Similarly, high CO_2_ fluxes, indicating greater microbial activity and carbon availability, only became significant drivers of N_2_O emissions under saturated conditions (Figure 2b). These observations align with the “hot moment” theory, where episodic high emissions arise from the simultaneous availability of substrates and suitable anaerobic conditions (Groffman et al., 2009; Krichels & Yang, 2019; Molodovskaya et al., 2012; Z. Zhang, Eddy, et al., 2024; Zentgraf et al., 2025). Soil NH_4_^+^ concentration was also a top predictor of N_2_O fluxes, with a sharp increase in predicted N_2_O emissions around 100 mg NH_4_^+^‐N (Table 2; Figure S4b). At higher NH_4_^+^ concentrations (>140 mg kg ^−1^), predicted emissions declined, likely reflecting limited data in that range and the tendency for such high NH_4_^+^ levels to occur under strongly saturated, reducing conditions where denitrification proceeds to N_2_ rather than N_2_O. Overall, these results reinforce that N_2_O emissions in this system respond nonlinearly to the joint availability of inorganic N, labile C, and soil moisture, with management and topography influencing emissions indirectly through their effects on these soil properties (Table S3).

### 3.2 Environmental drivers of methane emissions

**FIGURE 3:**
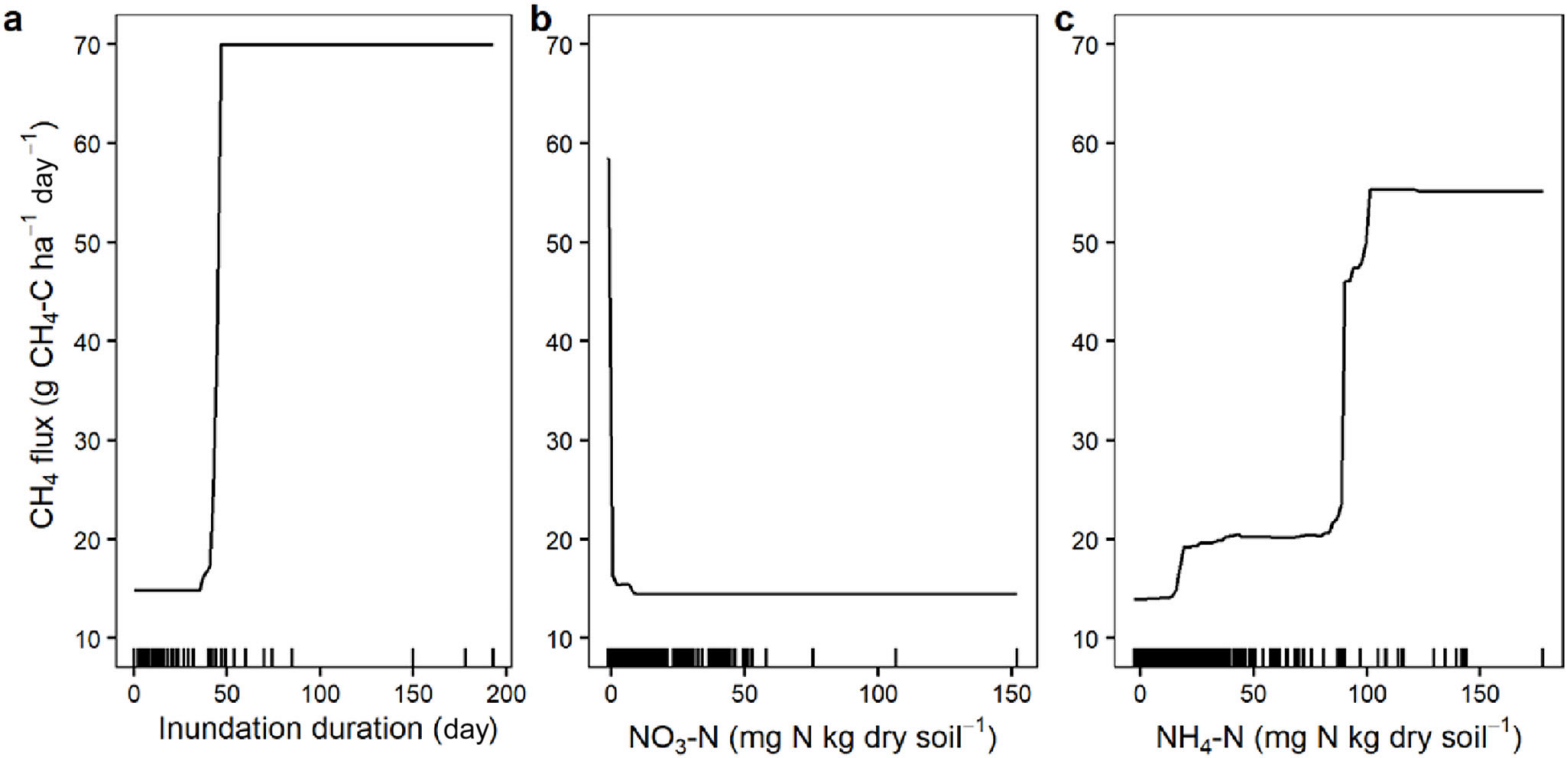
Top three predictor variables (>10% relative influence) for predicted CH_4_ flux from the CH_4_ boosted regression tree (BRT) model. Rug plots along the *x* axes show distribution of observed data.

For CH_4_ emissions, the BRT model identified inundation duration (i.e., the number of consecutive days soil moisture exceeded 50% VWC) as the most important predictor of emissions, followed by NO_3_^−^, NH_4_^+^, and CO_2_ flux (Table 2). Methane emissions remained low across most observations but increased sharply after approximately 40 consecutive days of saturation (Figures 1b and 3a). Once nitrate and oxygen were depleted, methanogenesis (preceded by possible iron reduction) became the dominant gas‐producing microbial pathway (Figure 1). In the BRT model, near‐zero NO_3_^−^ levels were associated with elevated CH_4_ fluxes, further suggesting NO_3_^−^ depletion marks a critical redox threshold favoring CH_4_ production (Figure 3b). Likewise, elevated NH_4_^+^ concentrations around ∼100 mg NH_4_^+^‐N also coincided with CH_4_ spikes (Figure 3c), which may reflect NH_4_^+^ accumulation from suppressed nitrification or recent manure application. As with N_2_O, topography and management had very little direct influence on CH_4_ emissions in the BRT model. This again suggests that GHG production is directly influenced by local redox conditions and substrate availability. The relatively low *R* ^2^ for the daily CH_4_ ANOVA model (*R* ^2^ = 0.18; Table 3) also reflects the episodic, threshold‐based nature of CH_4_ fluxes in upland systems, which are typically CH_4_ sinks except under prolonged anaerobic conditions (Brewer et al., 2018).

**TABLE 3:** Analysis of variance (ANOVA) results for daily and cumulative (annual) N_2_O and CH_4_ fluxes.

	Cumulative N_2_O		Cumulative CH_4_		Daily N_2_O			Daily CH_4_	
	*F*	*P*	*F*	*P*	*F*		*P*	*F*	*P*
Management	50.3	0.0001^***^	18.9	0.0001^***^	203.1	0.0001^***^		33.6	0.0001^***^
Topo	11.4	0.002^**^	9.5	0.04^*^	6.8	0.01^**^		30.5	0.0001^***^
Management × Topo	3.7	0.04^*^	1.9	0.2	5.3	0.01^**^		6.0	0.007^**^
Marginal *R* ^2^	0.80	–	0.94	–	0.70	–		0.18	–
Conditional *R* ^2^	–	–	–	–	0.70	–		0.18	–
*n*_observations_	36	–	36	–	1557	–		1557	–

*Note*: Topo, Topography.

^*^ *p* < 0.05. ^**^ *p* < 0.01. ^***^ *p* < 0.001.​

### 3.3 Influences of management and topography on nitrous oxide emissions

**FIGURE 4:**
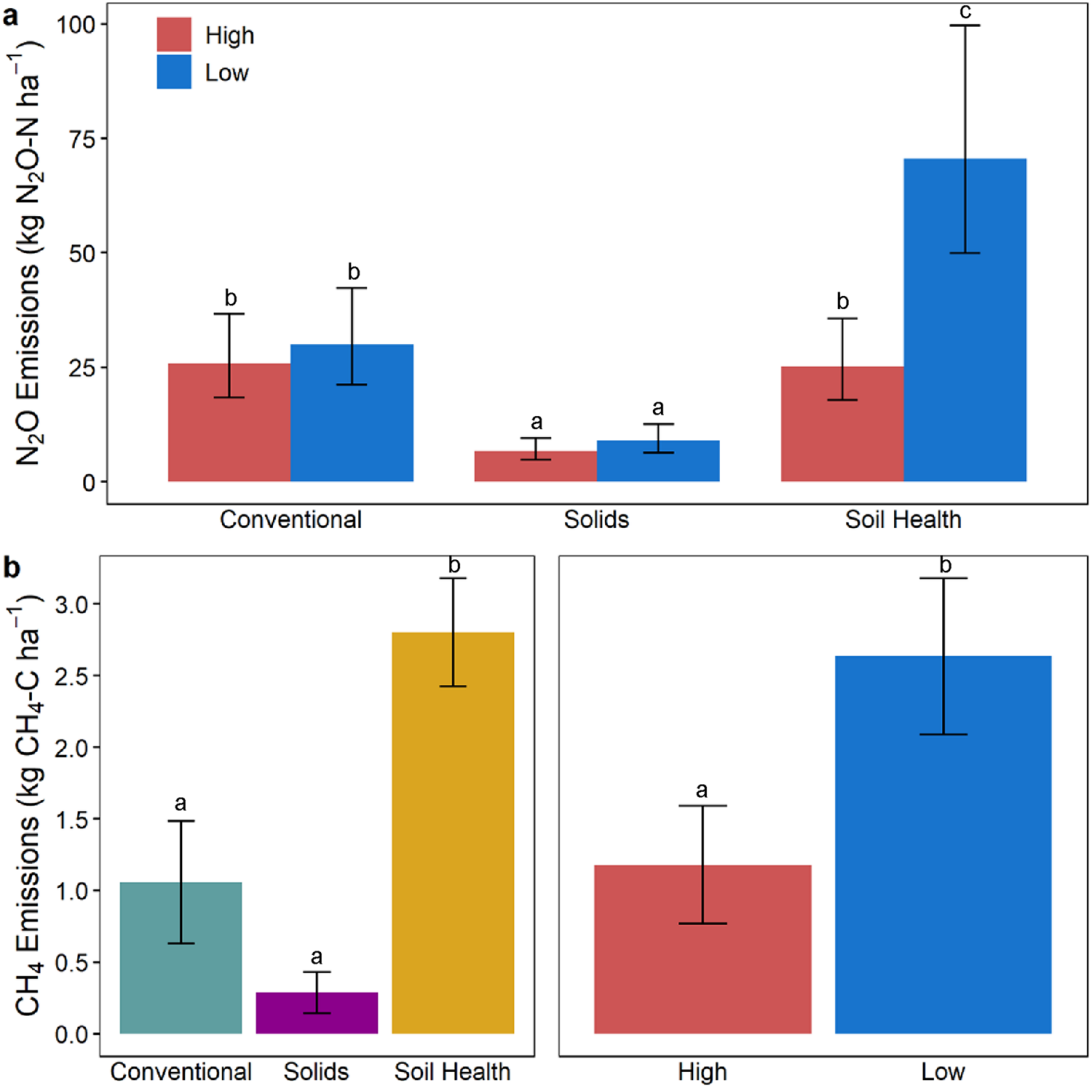
Cumulative N_2_O emissions (panel a) and CH_4_ emissions (panel b) from the first full year of treatment (October 6, 2023–October 6, 2024). Error bars indicate 95% confidence intervals for N_2_O (due to back‐transformation of log values) and mean ± standard error (SE) for CH_4_ emissions. Boxes that share a letter are not statistically different (*p* < 0.05) based on the Tukey post hoc test. Solids = Flocculated Solids.

Cumulative and daily N_2_O emissions differed by management, topographic position, and their interaction (*p* < 0.05; Table 3). Removing pretreatment observations did not affect the significance of management or topographic effects; however, the interaction was no longer significant for daily N_2_O fluxes (Table S4). Soil Health‐Low plots produced the highest annual N_2_O emissions (74.3 kg N_2_O‐N ha^−1^ year^−1^), significantly greater than all other treatment‐topography combinations (*p* < 0.05 Tukey HSD pairwise comparisons; Figure 4a), while Flocculated Solids plots exhibited the lowest, regardless of topographic position (Figure 4a). Daily N_2_O fluxes mirrored these cumulative trends (Table 3; Figure 1a). The highest of these values substantially exceeded typical cumulative emissions reported from upland row cropping systems in similar climates, which commonly range from 1 to 16 kg N_2_O‐N ha^−1^ year^−1^ (Cambareri et al., 2017; Dittmer et al., 2020; Lawrence et al., 2021), and likely reflect the convergence of site‐specific conditions, including poorly drained clay soils, record precipitation, and manure injection into wet, low areas of the field.

Elevated N_2_O emissions in Soil Health‐Low plots likely resulted from the combined influence of manure injection and saturated soil conditions. Injection can substantially reduce NH_3_ volatilization compared to surface application, retaining more inorganic N in the soil (Agnew et al., 2010; Duncan et al., 2017; Montes et al., 2013). Under prolonged wetness, such as in low areas of this field (Figure 1c), this retained N provided ample substrate for denitrification, producing higher N_2_O fluxes than in the Conventional system (Figure 1a), where surface‐applied manure likely lost more N to volatilization (Butterbach‐Bahl et al., 2013; Sherman et al., 2022). Soil inorganic N concentrations were also generally greater in low topographic positions (Figure 1d,e), further supporting greater nitrogen availability in these areas. Together, these results indicate that the higher emissions in the Soil Health‐Low plots arose from the environmental context of this management system—enhanced N retention and persistent wetness—rather than from the soil health management practices themselves (Figure 2; Table 2).

In contrast, the Flocculated Solids plots exhibited consistently lower N_2_O emissions. Because all manure treatments were applied on a P basis to avoid excessive P loading, the Flocculated Solids plots received less total N. Even when accounting for N input differences, greater emissions still occurred in the Soil Health plots. Uncorrected emission factors—calculated as the percent of applied N emitted as N_2_O‐N without background subtraction—averaged 10.5%, 13.3%, and 17.6% for the Conventional, Flocculated Solids, and Soil Health treatments, respectively. The intermediate emission factor for the Flocculated Solids indicates that reduced N_2_O fluxes cannot be attributed to lower N loading alone. Soil moisture, identified as a strong predictor of N_2_O fluxes in the BRT analysis (Table 2), differed primarily by topographic position and showed only marginal variation among management treatments (Table S3). Because management effects persisted in the BRT model after accounting for soil moisture, additional mechanisms beyond N input and minor moisture differences likely contributed to lower emissions observed in the Flocculated Solids plots. The solids were surface applied and had substantially higher dry matter content than the injected liquid manure (Table 1), resulting in less water added during application and a more stabilized organic N fraction with a higher C:N ratio, which likely influenced N placement, availability, and subsequent mineralization dynamics. Delayed N mineralization and temporary microbial N immobilization have been observed in other organic‐rich or stabilized manure amendments with relatively high C:N ratios (Biala et al., 2016; Gregorich et al., 2005; Xia et al., 2020), which likely contributed to lower N_2_O emissions in the Flocculated Solids plots.

### 3.4 Influences of management and topography on methane emissions

Cumulative CH_4_ emissions were influenced independently by treatment and topographic position, while daily CH_4_ emissions were influenced by their interaction (*p* < 0.05; Table 3 and Table S4). Low plots consistently emitted more CH_4_ than high plots, and Soil Health plots had the greatest overall emissions (*p* < 0.05 Tukey HSD pairwise comparisons; Figures 1b and 4b). Additionally, cumulative fluxes revealed that two individual chambers within Conventional‐High and Flocculated Solids‐High plots acted as net CH_4_ sinks, sequestering −60.3 and −40.6 g CH_4_‐C ha^−1^ year^−1^, respectively. However, these localized uptake patterns were outweighed by larger emission trends at the field scale.

Compared to the other two management strategies, greater CH_4_ emissions in Soil Health plots likely resulted from enhanced subsurface C availability and reduced oxygen diffusion in manure injection lines (Duncan et al., 2017; Sherlock et al., 2002; Figure S3a). Initial CH_4_ pulses measured upon manure injection could also reflect the release of stored CH_4_ already in the manure due to anaerobic storage conditions (Fangueiro et al., 2015; Wulf et al., 2002). Although the Conventional plots also received liquid manure, the combination of tillage and broadcast manure application in Conventional plots may have promoted methane oxidation through increased surface area at the soil–manure–air interface (Bayer et al., 2012; Fangueiro et al., 2015; O'Brien & Daigh, 2019). However, contrasting studies indicate that no‐till practices can instead enhance methane oxidation, suggesting that soil texture and moisture modulate these effects (Ball et al., 1999; Omonode et al., 2007; Ussiri et al., 2009).

Methane emissions from low plots were 2.2 times greater than high plot emissions (Figure 4b), reflecting significant differences in soil saturation and carbon availability (Tables SI and S3). Even though high areas of the field became saturated following rainfall, those effects were transient (Figure 1c). In contrast, certain low areas remained saturated for months at a time, resulting in different nutrient contents and redox conditions between topographic positions (Figure 1b,c; Table S1). Previous studies have confirmed that prolonged saturation is the primary driver of CH_4_ emissions in non‐rice agricultural systems (Hagedorn et al., 2022; Lehman & Osborne, 2013; Omonode et al., 2007), although research on CH_4_ emissions from upland row crops is still limited.

### 3.5 Temporal patterns of nitrous oxide and methane emissions

**FIGURE 5:**
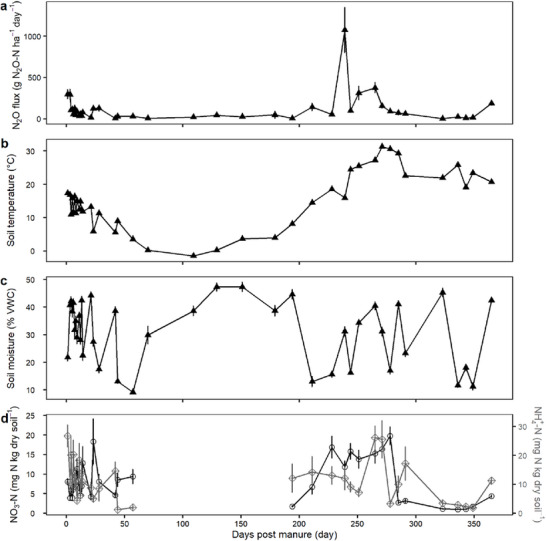
Mean daily N_2_O fluxes plotted across days after manure application. Data in each panel are averaged across all treatments. Symbol shapes distinguish variable types, with triangles representing N_2_O flux, soil temperature, and soil moisture. Inorganic N concentrations are shown with distinct symbols to differentiate nitrate (circles) and ammonium (diamonds). Because the exact 2022 manure‐application date was unknown, all sampling dates before October 5, 2023 were set to 365 days post‐manure. Soil samples for inorganic nitrogen were not taken during the winter months (panel d). VWC, volumetric water content.

Nitrous oxide fluxes exhibited strong seasonal dynamics, with the largest emissions observed in late spring and early summer in all treatments (Figure 1a). Despite the addition of nitrogen from fall manure application, N_2_O emissions remained comparatively low during the fall (Figure 1a). On average, fluxes during spring and summer were 1.7 times higher (177.0 ± 17.8 g N_2_O‐N ha^−1^ day^−1^) than those in the fall (104.0 ± 9.0 g N_2_O‐N ha^−1^ day^−1^). When N_2_O fluxes were examined by days post‐manure application (Figure 5a), distinct pulses were observed well beyond 200 days after application. These large springtime pulses likely resulted from a period of warming and drying that promoted nitrification of residual NH_4_^+^ (Figure 5d), followed by soil rewetting that triggered incomplete denitrification under partially anaerobic conditions (Figure 5a–c). Additionally, the largest N_2_O emission pulses in Spring 2024 occurred prior to the June sidedress application in the Conventional and Soil Health treatments (Figure 1a), indicating that these events were primarily triggered by environmental conditions rather than fertilizer addition. Similar springtime emissions have been reported in other studies and are often attributed to nitrate accumulation in low areas, minimal crop uptake early in the growing season, and abrupt spikes in soil temperature and moisture (Molodovskaya et al., 2012; Saha et al., 2017). These trends demonstrate that emission peaks were controlled less by the timing of manure addition and more by coinciding environmental conditions that simultaneously increased soil moisture and substrate availability.

Similar seasonal trends were observed for CH_4_ fluxes. In the warmer and wetter months before manure application, CH_4_ fluxes were 1.5 times greater than post‐manure fluxes. The prolonged period of soil saturation observed during July 2023–September 2023 likely contributed to these large pre‐manure application pulses (Figure 1b). Since this was before treatments were initiated, the pulses that occurred across all portions of the field were likely due exclusively to environmental conditions. However, smaller CH_4_ pulses were also present following manure application (Figure 1b), especially in the Soil Health treatment, likely due to pooled manure in subsurface injection slots.

### 3.6 Crop yield and N_2_O emissions intensity

Given that emissions were largely controlled by environmental context, particularly soil moisture, we examined whether these differences influenced crop production efficiency. Yield differed significantly by topographic position (*p* = 0.004; Table S5), with the highest yields in Soil Health‐High plots (33.9 ± 4.7 Mg ha^−1^; Table S6) and substantial declines in low plots across management systems. When expressed on a yield‐normalized basis, N_2_O intensity was greatest in low topographic positions (Table S6), with Soil Health‐Low plots exhibiting a fivefold increase relative to their high‐position counterparts. These findings suggest that poorly drained depressions function as inefficient production systems in wet years, generating a disproportionate climate cost per unit of crop produced. Although the Flocculated Solids plots exhibited consistently lower N_2_O intensities across topographic position, their P‐based nutrient management constraints limit broader applicability within Northeast dairy systems.

## 4 CONCLUSIONS

Together, these results highlight that GHG emissions reflect interactions among environmental factors—particularly soil moisture, carbon availability, and nitrogen pools. Practices like manure injection, which can enhance nitrogen retention, may also increase N_2_O emissions in moist, substrate‐rich areas of a field. Periods of extended saturation further promote denitrification and methanogenesis, especially in low‐lying, fine‐textured soils following heavy precipitation. Conversely, drier conditions and lower available nitrogen limited N_2_O emissions, as seen in plots receiving flocculated solids. These findings underscore the need for field‐specific, spatially informed management. Our results also identify key environmental thresholds beyond which N_2_O and CH_4_ emissions rise sharply, suggesting that tools like soil moisture sensors, nitrogen tests, and remote sensing could help flag high‐risk zones. As extreme rainfall becomes more common, management strategies must move beyond blanket prescriptions toward more spatially informed strategies that reduce emissions while maintaining productivity and healthy soils.

## AUTHOR CONTRIBUTIONS

**Molly Ratliff**: Data curation; formal analysis; investigation; writing—original draft; writing—review and editing. **Joshua W. Faulkner**: Conceptualization; funding acquisition; methodology; project administration; resources; supervision; writing—review and editing. **Eric D. Roy**: Resources; supervision; visualization; writing—review and editing. **Marie English**: Data curation; investigation; writing—review and editing. **Dan Liptzin**: Conceptualization; funding acquisition; methodology; project administration; writing—review and editing. **Reza K. Afshar**: Conceptualization; funding acquisition; project administration; writing—review and editing. **E. Carol Adair**: Conceptualization; data curation; formal analysis; funding acquisition; methodology; resources; supervision; visualization; writing—review and editing.

## CONFLICT OF INTEREST STATEMENT

The authors declare no conflicts of interest.

## Supporting information



Supplemental material on annual precipitation from the field site, initial soil properties across topography, field operations and management, relationships and ANOVA results for daily CO_2_ flux, soil moisture, soil temperature, and inorganic nitrogen; and partial dependence plots for predictors of nitrous oxide emissions from the BRT model.

## References

[jeq270187-bib-0001] Agnew, J. , Laguë, C. , Schoenau, J. , & Farrell, R. (2010). Greenhouse gas emissions measured 24 hours after surface and subsurface application of different manure types. Transactions of the ASABE, 53(5), 1689–1701. 10.13031/2013.34894

[jeq270187-bib-0002] Aronson, E. L. , & Helliker, B. R. (2010). Methane flux in non‐wetland soils in response to nitrogen addition: A meta‐analysis. Ecology, 91(11), 3242–3251. 10.1890/09-2185.1 21141185

[jeq270187-bib-0003] Ball, B. C. (2013). Soil structure and greenhouse gas emissions: A synthesis of 20 years of experimentation. European Journal of Soil Science, 64(3), 357–373. 10.1111/ejss.12013

[jeq270187-bib-0004] Ball, B. C. , Scott, A. , & Parker, J. P. (1999). Field N2O, CO2 and CH4 fluxes in relation to tillage, compaction and soil quality in Scotland. Soil and Tillage Research, 53(1), 29–39. 10.1016/S0167-1987(99)00074-4

[jeq270187-bib-0005] Bates, D. , Mächler, M. , Bolker, B. , & Walker, S. (2015). Fitting linear mixed‐effects models using lme4. Journal of Statistical Software, 67(1), 1–48. 10.18637/jss.v067.i01

[jeq270187-bib-0006] Bayer, C. , Gomes, J. , Vieira, F. C. B. , Zanatta, J. A. , de Cássia Piccolo, M. , & Dieckow, J. (2012). Methane emission from soil under long‐term no‐till cropping systems. Soil and Tillage Research, 124, 1–7. 10.1016/j.still.2012.03.006

[jeq270187-bib-0007] Biala, J. , Rowlings, D. , De Rosa, D. , & Grace, P. (2016). Effects of using raw and composted manures on nitrous oxide emissions: A review. Acta Horticulturae, 1112, 425–430. 10.17660/ActaHortic.2016.1112.57

[jeq270187-bib-0008] Borchers, H. W. (2022). pracma: Practical Numerical Math Functions (R Package Version 2.3.8) [Computer software]. CRAN. https://CRAN.R‐project.org/package=pracma

[jeq270187-bib-0009] Brewer, P. E. , Calderón, F. , Vigil, M. , & von Fischer, J. C. (2018). Impacts of moisture, soil respiration, and agricultural practices on methanogenesis in upland soils as measured with stable isotope pool dilution. Soil Biology and Biochemistry, 127, 239–251. 10.1016/j.soilbio.2018.09.014

[jeq270187-bib-0010] Brickman, S. , Darby, H. , Ruhl, L. , & Adair, E. C. (2024). Nitrous oxide emissions are driven by environmental conditions rather than nitrogen application methods in a perennial hayfield. Journal of Environmental Quality, 53(2), 133–146. 10.1002/jeq2.20536 38127325

[jeq270187-bib-0011] Bubier, J. , Costello, A. , Moore, T. R. , Roulet, N. T. , & Savage, K. (1993). Microtopography and methane flux in boreal peatlands, northern Ontario, Canada. Canadian Journal of Botany, 71(8), 1056–1063. 10.1139/b93-122

[jeq270187-bib-0012] Butterbach‐Bahl, K. , Baggs, E. M. , Dannenmann, M. , Kiese, R. , & Zechmeister‐Boltenstern, S. (2013). Nitrous oxide emissions from soils: How well do we understand the processes and their controls? Philosophical Transactions of the Royal Society B: Biological Sciences, 368(1621), 20130122. 10.1098/rstb.2013.0122 PMC3682742 23713120

[jeq270187-bib-0013] Cambareri, G. , Drury, C. , Lauzon, J. , Salas, W. , & Wagner‐Riddle, C. (2017). Year‐round nitrous oxide emissions as affected by timing and method of dairy manure application to corn. Soil Science Society of America Journal, 81(1), 166–178. 10.2136/sssaj2016.05.0160

[jeq270187-bib-0014] Chan, A. S. K. , & Parkin, T. B. (2001). Methane Oxidation and production activity in soils from natural and agricultural ecosystems. Journal of Environmental Quality, 30(6), 1896–1903. 10.2134/jeq2001.1896 11789994

[jeq270187-bib-0015] Chapuis‐Lardy, L. , Wrage, N. , Metay, A. , Chotte, J.‐L. , & Bernoux, M. (2007). Soils, a sink for N_2_O? A review. Global Change Biology, 13(1), 1–17. 10.1111/j.1365-2486.2006.01280.x

[jeq270187-bib-0016] Cloutier, M. L. , Liptzin, D. , Coyotl, A. , Baxter, A. E. , & Morgan, C. L. S. (2025). Environmental sustainability in US dairy farms: Policies, practices, and outcomes. Journal of Environmental Quality, 54, 1163–1186. 10.1002/jeq2.70031 40329760 PMC12431966

[jeq270187-bib-0018] Dittmer, K. M. , Darby, H. M. , Goeschel, T. R. , & Adair, E. C. (2020). Benefits and tradeoffs of reduced tillage and manure application methods in a *Zea mays* silage system. Journal of Environmental Quality, 49(5), 1236–1250. 10.1002/jeq2.20125 33016461

[jeq270187-bib-0019] Duncan, E. W. , Dell, C. J. , Kleinman, P. J. , & Beegle, D. B. (2017). Nitrous oxide and ammonia emissions from injected and broadcast‐applied dairy slurry. Journal of Environmental Quality, 46(1), 36–44. 10.2134/jeq2016.05.0171 28177424

[jeq270187-bib-0020] Elberling, B. B. , Kovács, G. M. , Hansen, H. F. E. , Fensholt, R. , Ambus, P. , Tong, X. , Gominski, D. , Mueller, C. W. , Poultney, D. M. N. , & Oehmcke, S. (2023). High nitrous oxide emissions from temporary flooded depressions within croplands. Communications Earth & Environment, 4(1), Article 463. 10.1038/s43247-023-01095-8

[jeq270187-bib-0021] Elith, J. , Leathwick, J. R. , & Hastie, T. (2008). A working guide to boosted regression trees. Journal of Animal Ecology, 77(4), 802–813. 10.1111/j.1365-2656.2008.01390.x 18397250

[jeq270187-bib-0022] Fangueiro, D. , Surgy, S. , Fraga, I. , Cabral, F. , & Coutinho, J. (2015). Band application of treated cattle slurry as an alternative to slurry injection: Implications for gaseous emissions, soil quality, and plant growth. Agriculture, Ecosystems & Environment, 211, 102–111. 10.1016/j.agee.2015.06.003

[jeq270187-bib-0023] Forester, J. C. (1995). Soil nitrogen. In K. Alef & P. Nannipieri (Eds.), Methods in applied soil microbiology and biochemistry (pp. 79–87). Academic Press.

[jeq270187-bib-0024] Grave, R. A. , da Silveira Nicoloso, R. , Cassol, P. C. , da Silva, M. L. B. , Mezzari, M. P. , Aita, C. , & Wuaden, C. R. (2018). Determining the effects of tillage and nitrogen sources on soil N_2_O emission. Soil and Tillage Research, 175, 1–12. 10.1016/j.still.2017.08.011

[jeq270187-bib-0025] Greenwell, B. , Boehmke, B. , Cunningham, J. , & Developers, G. B. M. (2020). gbm: Generalized Boosted Regression Models (R Package Version 2.1.8) [Computer software]. CRAN. https://CRAN.R‐project.org/package=gbm

[jeq270187-bib-0026] Gregorich, E. G. , Rochette, P. , VandenBygaart, A. J. , & Angers, D. A. (2005). Greenhouse gas contributions of agricultural soils and potential mitigation practices in Eastern Canada. Soil and Tillage Research, 83(1), 53–72. 10.1016/j.still.2005.02.009

[jeq270187-bib-0027] Groffman, P. , Butterbach‐Bahl, K. , Fulweiler, W. , Gold, A. , Morse, J. , Stander, E. , Tague, C. , Tonitto, C. , & Vidon, P. (2009). Incorporating spatially and temporally explicit phenomena (hotspots and hot moments) in denitrification models. Biogeochemistry, 93, 49–77. 10.1007/s10533-008-9277-5

[jeq270187-bib-0028] Gu, J. , Nicoullaud, B. , Rochette, P. , Pennock, D. J. , Hénault, C. , Cellier, P. , & Richard, G. (2011). Effect of topography on nitrous oxide emissions from winter wheat fields in Central France. Environmental Pollution, 159(11), 3149–3155. 10.1016/j.envpol.2011.04.009 21531057

[jeq270187-bib-0029] Guo, X. , Drury, C. F. , Yang, X. , Reynolds, D. , & Fan, R. (2014). The extend of soil drying and rewetting affects nitrous oxide emissions, denitrification, and nitrogen mineralization. Soil Science Society of America, 78(1), 194–204. 10.2136/sssaj2013.06.0219

[jeq270187-bib-0030] Hagedorn, J. G. , Davidson, E. A. , Fisher, T. R. , Fox, R. J. , Zhu, Q. , Gustafson, A. B. , Koontz, E. , Castro, M. S. , & Lewis, J. (2022). Effects of drainage water management in a corn–soy rotation on soil N_2_O and CH_4_ Fluxes. Nitrogen, 3(1), 128–148. https://www.mdpi.com/2504‐3129/3/1/10

[jeq270187-bib-0031] Hansen, L. V. , Brændholt, A. , Tariq, A. , Jensen, L. S. , Peixoto, L. E. K. , Petersen, S. O. , & Bruun, S. (2024). Methane uptake rates across different soil types and agricultural management practices in Denmark. Agriculture, Ecosystems & Environment, 363, 108878. 10.1016/j.agee.2023.108878

[jeq270187-bib-0032] Hatano, R. , Mukumbuta, I. , & Shimizu, M. (2024). Soil health intensification through strengthening soil structure improves soil carbon sequestration. Agriculture, 14(8), 1290. https://www.mdpi.com/2077‐0472/14/8/1290

[jeq270187-bib-0033] Hicke, J. A. , Lucatello, S. , Mortsch, L. D. , Dawson, J. , Dominguez Aguilar, M. , Enquist, C. A. F. , Gilmore, E. A. , Gutzler, D. S. , Harper, S. , Holsman, K. , Jewett, E. B. , Kohler, T. A. , & Miller, K. A. (2022). *Climate change 2022: Impacts, adaptation and vulnerability* (Contribution of Working Group II to the Sixth Assessment Report of the Intergovernmental Panel on Climate Change: H.‐O. Pörtner , D. C. Roberts , M. Tignor , E. S. Poloczanska , K. Mintenbeck , A. Alegría , M. Craig , S. Langsdorf , S. Löschke , V. Möller , A. Okem , & B. Rama [Eds.]). Cambridge University Press. 10.1017/9781009325844.016

[jeq270187-bib-0034] Intergovernmental Panel on Climate Change (IPCC) . (2013). *Climate change 2013: The physical science basis* (Contribution of Working Group I to the Fifth Assessment Report of the Intergovernmental Panel on Climate Change: T. F. Stocker , D. Qin , G.‐K. Plattner , M. Tignor , S. K. Allen , J. Boschung , A. Nauels , Y. Xia , V. Bex , & P. M. Midgley [Eds.]). Cambridge University Press. 10.1017/CBO9781107415324

[jeq270187-bib-0035] Intergovernmental Panel on Climate Change (IPCC) . (2014). Climate change 2014: Synthesis report (Contribution of Working Groups I, II and III to the Fifrth Assessment Report of the Intergovernmental Panel on Climate Change, Core Writing Team: R. K. Pachauri & L. A. Meyer (Eds.)). IPCC.

[jeq270187-bib-0036] Krichels, A. , DeLucia, E. H. , Sanford, R. , Chee‐Sanford, J. , & Yang, W. H. (2019). Historical soil drainage mediates the response of soil greenhouse gas emissions to intense precipitation events. Biogeochemistry, 142(3), 425–442. 10.1007/s10533-019-00544-x

[jeq270187-bib-0037] Krichels, A. H. , & Yang, W. H. (2019). Dynamic Controls on field‐scale soil nitrous oxide hot spots and hot moments across a microtopographic gradient. Journal of Geophysical Research: Biogeosciences, 124(11), 3618–3634. 10.1029/2019JG005224

[jeq270187-bib-0038] Kuhn, M. (2022). caret: Classification and Regression Training (R Package Version 6.0‐91) [Computer software]. CRAN. https://CRAN.R‐project.org/package=caret

[jeq270187-bib-0039] Lawrence, N. C. , Tenesaca, C. G. , VanLoocke, A. , & Hall, S. J. (2021). Nitrous oxide emission from agricultural soils challenge climate sustainability in the US Corn Belt. Proceedings of the National Academy of Science of the USA, 118(46), e2112108118. 10.1073/pnas.2112108118 PMC8694048 34750266

[jeq270187-bib-0040] Lehman, R. M. , & Osborne, S. L. (2013). Greenhouse gas fluxes from no‐till rotated corn in the upper Midwest. Agriculture, Ecosystems & Environment, 170, 1–9. 10.1016/j.agee.2013.02.009

[jeq270187-bib-0041] Lehmann, J. , Bossio, D. A. , Kögel‐Knabner, I. , & Rillig, M. C. (2020). The concept and future prospects of soil health. Nature Reviews Earth & Environment, 1(10), 544–553. 10.1038/s43017-020-0080-8 PMC7116140 33015639

[jeq270187-bib-0043] Li, Y. , Shen, Y. , & Wang, T. (2021). Freezing and thawing cycles affect nitrous oxide emissions in rain‐fed lucerne (*Medicago sativa)* grasslands of different ages. PeerJ, 9, e12216. 10.7717/peerj.12216 34707931 PMC8501990

[jeq270187-bib-0044] LI‐COR Biosciences . (n.d.). Measurement programming and data analysis . https://www.licor.com/support/Smart‐Chamber/topics/trace‐gas‐sample‐kit‐data‐processing.html?Highlight=static%20chamber%20flux%20equation

[jeq270187-bib-0045] Liu, H. , Zheng, X. , Li, Y. , Yu, J. , Ding, H. , Sveen, T. R. , & Zhang, Y. (2022). Soil moisture determines nitrous oxide emission and uptake. Science of the Total Environment, 822, 153566. 10.1016/j.scitotenv.2022.153566 35104523

[jeq270187-bib-0047] Maljanen, M. , Liikanen, A. , Silvola, J. , & Martikainen, P. (2006). Methane fluxes on agricultural and forested boreal organic soils. Soil Use and Management, 19, 73–79. 10.1111/j.1475-2743.2003.tb00282.x

[jeq270187-bib-0048] Molodovskaya, M. , Singurindy, O. , Richards, B. K. , Warland, J. , Johnson, M. S. , & Steenhuis, T. S. (2012). Temporal variability of nitrous oxide from fertilized croplands: Hot moment analysis. Soil Science Society of America Journal, 76(5), 1728–1740. 10.2136/sssaj2012.0039

[jeq270187-bib-0049] Montes, F. , Meinen, R. , Dell, C. , Rotz, A. , Hristov, A. N. , Oh, J. , Waghorn, G. , Gerber, P. J. , Henderson, B. , Makkar, H. P. , & Dijkstra, J. (2013). Special topics—Mitigation of methane and nitrous oxide emissions from animal operations: II. A review of manure management mitigation options. Journal of Animal Science, 91(11), 5070–5094. 10.2527/jas.2013-6584 24045493

[jeq270187-bib-0050] Murphy, P. N. C. , Ogilvie, J. , & Arp, P. (2009). Topographic modelling of soil moisture conditions: A comparison and verification of two models. European Journal of Soil Science, 60(1), 94–109. 10.1111/j.1365-2389.2008.01094.x

[jeq270187-bib-0051] Natural Resources Conservation Service . (2023). Practice specification: Nutrient management (Code 590) (590‐PS‐1) . U.S. Department of Agriculture. https://www.nrcs.usda.gov/sites/default/files/2023‐06/Practice%20Specification%20Nutrient%20Management%20%28Code%20590%29.pdf

[jeq270187-bib-0052] Negassa, W. , Price, R. F. , Basir, A. , Snapp, S. S. , & Kravchenko, A. (2015). Cover crop and tillage systems effect on soil CO_2_ and N_2_O fluxes in contrasting topographic positions. Soil and Tillage Research, 154, 64–74. 10.1016/j.still.2015.06.015

[jeq270187-bib-0053] O'Brien, P. L. , & Daigh, A. L. M. (2019). Tillage practices alter the surface energy balance—A review. Soil and Tillage Research, 195, 104354. 10.1016/j.still.2019.104354

[jeq270187-bib-0054] O'Brien, P. L. , Emmett, B. D. , Malone, R. W. , Nunes, M. R. , Kovar, J. L. , Kaspar, T. C. , Moorman, T. B. , Jaynes, D. B. , & Parkin, T. B. (2022). Nitrate losses and nitrous oxides emissions under contrasting tillage and cover crop management. Journal of Environmental Quality, 51(4), 683–695. 10.1002/jeq2.20361 35443288

[jeq270187-bib-0055] Oertel, C. , Matschullat, J. , Zurba, K. , Zimmermann, F. , & Erasmi, S. (2016). Greenhouse gas emissions from soils—A review. Geochemistry, 76(3), 327–352. 10.1016/j.chemer.2016.04.002

[jeq270187-bib-0056] Omonode, R. A. , Vyn, T. J. , Smith, D. R. , Hegymegi, P. , & Gál, A. (2007). Soil carbon dioxide and methane fluxes from long‐term tillage systems in continuous corn and corn–soybean rotations. Soil and Tillage Research, 95(1), 182–195. 10.1016/j.still.2006.12.004

[jeq270187-bib-0057] Pärn, J. , Verhoeven, J. T. A. , Butterbach‐Bahl, K. , Dise, N. B. , Ullah, S. , Aasa, A. , Egorov, S. , Espenberg, M. , Järveoja, J. , Jauhiainen, J. , Kasak, K. , Klemedtsson, L. , Kull, A. , Laggoun‐Défarge, F. , Lapshina, E. D. , Lohila, A. , Lõhmus, K. , Maddison, M. , Mitsch, W. J. , & Mander, Ü. (2018). Nitrogen‐rich organic soils under warm well‐drained conditions are global nitrous oxide emission hotspots. Nature Communications, 9(1), Article 1135. 10.1038/s41467-018-03540-1 PMC5859301 29555906

[jeq270187-bib-0058] Paustian, K. , Lehmann, J. , Ogle, S. , Reay, D. , Robertson, G. P. , & Smith, P. (2016). Climate‐smart soils. Nature, 532, 49–57. 10.1038/nature17174 27078564

[jeq270187-bib-0059] Perryman, C. R. , McCalley, C. K. , Ernakovich, J. G. , Lamit, L. J. , Shorter, J. H. , Lilleskov, E. , & Varner, R. K. (2022). Microtopography matters: Belowground CH_4_ cycling regulated by differing microbial processes in peatland hummocks and lawns. Journal of Geophysical Research: Biogeosciences, 127(8), e2022JG006948. 10.1029/2022JG006948

[jeq270187-bib-0060] Ponce de León, M. A. , Dell, C. J. , & Karsten, H. D. (2021). Nitrous oxide emissions from manured, no‐till corn systems. Nutrient Cycling in Agroecosystems, 119, 405–421. 10.1007/s10705-021-10131-y

[jeq270187-bib-0061] Porterfield, K. K. , Faulkner, J. , & Roy, E. D. (2020). Nutrient recovery from anaerobically digested dairy manure using dissolved air flotation (DAF). ACS Sustainable Chemistry & Engineering, 8(4), 1964–1970. 10.1021/acssuschemeng.9b06419

[jeq270187-bib-0062] Pugesgaard, S. , Petersen, S. O. , Chirinda, N. , & Olesen, J. E. (2017). Crop residues as driver for N_2_O emissions from a sandy loam soil. Agricultural and Forest Meteorology, 233, 45–54. 10.1016/j.agrformet.2016.11.007

[jeq270187-bib-0063] Regina, K. , & Alakukku, L. (2010). Greenhouse gas fluxes in varying soils types under conventional and no‐tillage practices. Soil and Tillage Research, 109(2), 144–152. 10.1016/j.still.2010.05.009

[jeq270187-bib-0064] RStudio Team . (2022). RStudio: Integrated development environment for R . RStudio, PBC. http://www.rstudio.com/

[jeq270187-bib-0066] Saha, D. , Rau, B. M. , Kaye, J. P. , Montes, F. , Adler, P. R. , & Kemanian, A. R. (2017). Landscape control of nitrous oxide emissions during the transition from conservation reserve program to perennial grasses for bioenergy. GCB Bioenergy, 9(4), 783–795. 10.1111/gcbb.12395

[jeq270187-bib-0067] Sánchez‐Rodríguez, A. R. , Nie, C. , Hill, P. W. , Chadwick, D. R. , & Jones, D. L. (2019). Extreme flood events at higher temperatures exacerbate the loss of soil functionality and trace gas emissions in grassland. Soil Biology and Biochemistry, 130, 227–236. 10.1016/j.soilbio.2018.12.021

[jeq270187-bib-0068] Schaufler, G. , Kitzler, B. , Schindlbacher, A. , Skiba, U. , Sutton, M. A. , & Zechmeister‐Boltenstern, S. (2010). Greenhouse gas emissions from European soils under different land use: Effects of soil moisture and temperature. European Journal of Soil Science, 61(5), 683–696. 10.1111/j.1365-2389.2010.01277.x

[jeq270187-bib-0069] Scott, B. , Baldwin, A. H. , & Yarwood, S. A. (2022). Quantification of potential methane emissions associated with organic matter amendments following oxic‐soil inundation. Biogeosciences, 19(4), 1151–1164. 10.5194/bg-19-1151-2022

[jeq270187-bib-0070] Sherlock, R. R. , Sommer, S. G. , Khan, R. Z. , Wood, C. W. , Guertal, E. A. , Freney, J. R. , Dawson, C. O. , & Cameron, K. C. (2002). Ammonia, methane, and nitrous oxide emission from pig slurry applied to a pasture in New Zealand. Journal of Environmental Quality, 31(5), 1491–1501. 10.2134/jeq2002.1491 12371166

[jeq270187-bib-0071] Sherman, J. , Young, E. , Jokela, W. , & Kieke, B. (2022). Manure application timing and incorporation effects on ammonia and greenhouse gas emissions in corn. Agriculture, 12(11), 1952. https://www.mdpi.com/2077‐0472/12/11/1952

[jeq270187-bib-0072] Shukla, T. , Tang, W. , Trettin, C. C. , Chen, G. , Chen, S. , & Allan, C. (2023). Quantification of microtopography in natural ecosystems using close‐range remote sensing. Remote Sensing, 15(9), 2387. https://www.mdpi.com/2072‐4292/15/9/2387

[jeq270187-bib-0073] Smith, P. , Reay, D. , & Smith, J. (2021). Agricultural methane emissions and the potential for mitigation. Philosophical Transactions of the Royal Society A: Mathematical, Physical and Engineering Sciences, 379(2210), 20200451. 10.1098/rsta.2020.0451 34565225

[jeq270187-bib-0074] Suriyavirun, N. , Krichels, A. H. , Kent, A. D. , & Yang, W. H. (2019). Microtopographic differences in soil properties and microbial community composition at the field scale. Soil Biology and Biochemistry, 131, 71–80. 10.1016/j.soilbio.2018.12.024

[jeq270187-bib-0075] Syakila, A. , & Kroeze, C. (2011). The global nitrous oxide budget revisited. Greenhouse Gas Measurement and Management, 1(1), 17–26. 10.3763/ghgmm.2010.0007

[jeq270187-bib-0076] United States Department of Agriculture Economic Research Service . (2025). Land use, land value & tenure . https://www.ers.usda.gov/topics/farm‐economy/land‐use‐land‐value‐tenure

[jeq270187-bib-0077] Univeristy of Vermont Extension . (2018). Nutrient recommendations for field crops in Vermont (BR 1390.2). https://www.uvm.edu/d10‐files/documents/2024‐08/NutrientRecs_BR1390.2.pdf

[jeq270187-bib-0078] Ussiri, D. A. N. , Lal, R. , & Jarecki, M. K. (2009). Nitrous oxide and methane emissions from long‐term tillage under a continuous corn cropping system in Ohio. Soil and Tillage Research, 104(2), 247–255. 10.1016/j.still.2009.03.001

[jeq270187-bib-0079] van Kessel, C. , Venterea, R. , Six, J. , Adviento‐Borbe, M. A. , Linquist, B. , & van Groenigen, K. J. (2013). Climate, duration, and N placement determine N_2_O emissions in reduced tillage systems: A meta‐analysis. Global Change Biology, 19(1), 33–44. 10.1111/j.1365-2486.2012.02779.x 23504719

[jeq270187-bib-0080] Vermont Agency of Agriculture, Food & Markets . (n.d.). Nutrient management planning (NMP) . https://agriculture.vermont.gov/nmp

[jeq270187-bib-0081] Vilain, G. , Garnier, J. , Tallec, G. , & Cellier, P. (2010). Effect of slope position and land use on nitrous oxide (N_2_O) emissions (Seine Basin, France). Agricultural and Forest Meteorology, 150(9), 1192–1202. 10.1016/j.agrformet.2010.05.004

[jeq270187-bib-0082] Walthall, C. L. , Hatfield, J. , Backlund, P. , Lengnick, L. , Marshall, E. , Walsh, M. , Adkins, S. , Aillery, M. , Ainsworth, E. A. , Ammann, C. , Anderson, C. J. , Bartomeus, I. , Baumgard, L. H. , Booker, F. , Bradley, B. , Blumenthal, D. M. , Bunce, J. , Burkey, K. , Dabney, S. M. , … Ziska, L. H. (2012). Climate change and agriculture in the United States: Effects and adaptation (Technical Bulletin 1935). USDA.

[jeq270187-bib-0083] Wang, C. , Amon, B. , Schulz, K. , & Mehdi, B. (2021). Factors that influence nitrous oxide emissions from agricultural soils as well as their representation in simulation models: A review. Agronomy, 11(4), 770. https://www.mdpi.com/2073‐4395/11/4/770

[jeq270187-bib-0084] Wang, M. , Liu, H. , Rezanezhad, F. , Zak, D. , & Lennartz, B. (2023). The influence of microtopography on soil carbon accumulation and nutrient release from a rewetted coastal peatland. Geoderma, 438, 116637. 10.1016/j.geoderma.2023.116637

[jeq270187-bib-0085] White, A. , Darby, H. , Ruhl, L. , & Sands, B. (2023). Long term influence of alternative corn cropping practices and corn‐hay rotations on soil health, yields and forage quality. Frontiers in Environmental Science, 11, 1061013. 10.3389/fenvs.2023.1061013

[jeq270187-bib-0086] Wulf, S. , Maeting, M. , & Clemens, J. (2002). Application technique and slurry co‐fermentation effects on ammonia, nitrous oxide, and methane emissions after spreading. Journal of Environmental Quality, 31(6), 1795–1801. 10.2134/jeq2002.1795 12469828

[jeq270187-bib-0087] Xia, F. , Mei, K. , Xu, Y. , Zhang, C. , Dahlgren, R. A. , & Zhang, M. (2020). Response of N_2_O emission to manure application in field trials of agricultural soils across the globe. Science of the Total Environment, 733, 139390. 10.1016/j.scitotenv.2020.139390 32446092

[jeq270187-bib-0088] Zentgraf, I. , Holz, M. , Monzón Díaz, O. R. , Lück, M. , Kramp, K. , Pusch, V. , Grahmann, K. , & Hoffmann, M. (2025). How scale affects N_2_O emissions in heterogeneous fields of a diversified agricultural landscape. Scientific Reports, 15(1), Article 11013. 10.1038/s41598-025-95630-6 40164655 PMC11958750

[jeq270187-bib-0089] Zhang, M. , Fu, L. , Ma, D. , Wang, X. , & Liu, A. (2024). Effects of microtopography on soil microbial community structure and abundance in permafrost peatlands. Microorganisms, 12(5), 867. 10.3390/microorganisms12050867 38792697 PMC11124213

[jeq270187-bib-0090] Zhang, Z. , Eddy, W. C. , Stuchiner, E. R. , DeLucia, E. H. , & Yang, W. H. (2024). A conceptual model explaining spatial variation in soil nitrous oxide emissions in agricultural fields. Communications Earth & Environment, 5(1), Article 730. 10.1038/s43247-024-01875-w

[jeq270187-bib-0091] Zheng, X. , Wang, M. , Wang, Y. , Shen, R. , Gou, J. , Li, J. , Jin, J. , & Li, L. (2000). Impacts of soil moisture on nitrous oxide emission from croplands: A case study on the rice‐based agro‐ecosystem in Southeast China. Chemosphere—Global Change Science, 2(2), 207–224. 10.1016/S1465-9972(99)00056-2

