# Supplementary material for: Topography, management, and extreme precipitation influence greenhouse gas emissions in a cool, humid corn silage system
Source: J Environ Qual. 2026 Apr 29;55:e70187. doi: 10.1002/jeq2.70187 (PMC13128991; doi:10.1002/jeq2.70187)
Supplement: Supplementary file 1 — Supplemental material on annual precipitation from the field site, initial soil properties across topography, field operations and management, relationships and ANOVA results for daily CO2 flux, soil moisture, soil temperature, and inorganic nitrogen; and partial dependence plots for predictors of nitrous oxide emissions from the BRT model. [file JEQ2-55-0-s001.docx]

**Supplemental Material**

Topography, management, and extreme precipitation influence greenhouse gas emissions in a cool, humid corn silage system

Molly Ratliff, Joshua Faulkner, Eric D. Roy, Marie English, Dan Liptzin, Reza K. Afshar, E. Carol Adair

List of Supplemental Tables and Figures

Supplemental Figure S1. Cumulative precipitation for 2023 and 2024.

Supplemental Table SI. Initial soil properties averaged across topographic high and topographic low plots.

Supplemental Figure S2. Schematic diagram of the experimental field.

Supplemental Table SII. Summary of field operations and management activities

Supplemental Figure S3. Relationships between nitrous oxide emissions and soil moisture, soil temperature, soil nitrate concentrations, and soil ammonium concentrations.

**Supplemental Table SIII.** Analysis of variance (ANOVA) results for daily CO_2_ fluxes, soil moisture, soil ammonium concentrations, and soil nitrate concentrations.

**Supplemental Figure S4.** Partial dependence plots for the top four predictor variables of N_2_O flux in the best iteration of the N_2_O BRT model.

**Supplemental Table SIV.** Analysis of variance (ANOVA) results for daily N_2_O and CH_4_ emissions using post-treatment observations only.

**Supplemental Table SV.** Analysis of variance (ANOVA) results for 2024 corn silage yield and N_2_O intensity.

**Supplemental Table SVI.**  Mean (**±**SE) 2024 corn silage yield (ton ha^-1^) and N_2_O intensity (kg N2O-N per ton corn) by management treatment and topographic position.

**Supplemental Figure S1.** Cumulative precipitation for 2023 (blue line) and 2024 (red line) at the field site compared to the averaged 30-year normal (black line). Historic precipitation data and 2023 data retrieved using the *daymetr* R package, and 2024 precipitation measured at the field.


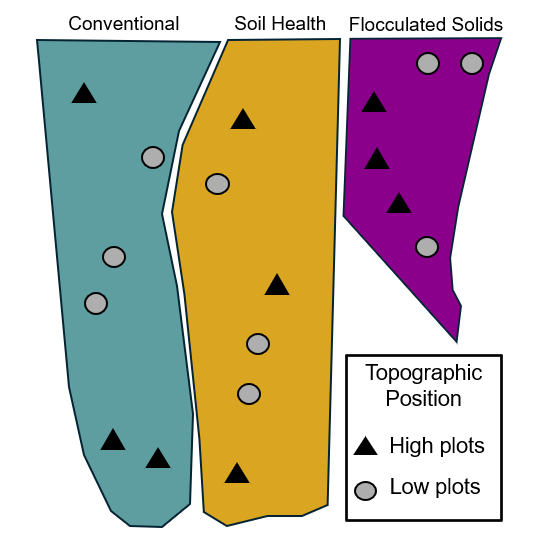


**Supplemental Figure S2.** Schematic representation of the experimental field showing the three management treatments blocks and greenhouse gas sampling plots by topographic position. This diagram is not to scale.

**SUPPLEMENTAL TABLE SI** Summary of initial soil properties divided by topography (June 2023, 0-15cm)

| **Soil Property** | **Low Topography** | **High Topography** |
| --- | --- | --- |
| Total Carbon (%) | 3.4 ± 0.8 | 2.7 ± 0.6 |
| Total Nitrogen (%) | 0.3 ± 0.07 | 0.3 ± 0.05 |
| NH_4_^+^-N (mg kg^-1^) | 11.4 ± 3.0 | 8.4 ± 2.6 |
| NO_3_^-^-N (mg kg^-1^) | 28.7 ± 10.3 | 30.5 ± 6.1 |
| Soil pH | 6.3 ± 0.5 | 6.03 ± 0.4 |
| Bulk Density (g cm^-3^) | 1.0 ± 0.2 | 1.2 ± 0.1 |

**SUPPLEMENTAL TABLE SII** Summary of field operations and management activities. All field operations were managed using typical field-scale equipment. Solids = Flocculated Solids

| **Event** | **Treatment** | **Date(s)** | **Method** | **Rate & Notes** |
| --- | --- | --- | --- | --- |
| **2023** | | | | |
| Spring tillage | Conventional | May 16, 2023 | Chisel plow | 15 cm depth |
| Corn planting | All | May 16, 2023 | No-till (Soil Health, Solids); conventional |  |
| Cover crop termination | Soil Health, Solids | May 17, 2023 | Glyphosate application | Standard rate |
| Sidedress | All | Late June | Broadcast urea pellets | 200 lbs acre^-1^ (38-0-0; ~ 85 kg N ha^-1^) |
| Harvest | All | Sep 25, 2023 | Chopped for silage | Forage harvester with yield monitor |
| Manure application | All | Oct 5, 2023 | Broadcast (Conventional), injection (Soil Health), manure spreader (Solids) | See Table 1 |
| Cover crop planting | Soil Health, Solids | Oct 20, 2023 | 30 ft Grain drill | 80 lbs/ac |
| **2024** | | | | |
| Spring tillage | Conventional | May 14, 2024 | Chisel plow | 15 cm depth |
| Corn planting | All | May 11, May 14, 2024 | No-till (Soil Health, Solids); conventional |  |
| Cover crop termination | Soil Health, Solids | May 17, 2024 | Glyphosate application | Standard rate |
| Sidedress | All | Jun 19, 2024 | Broadcast urea pellets | 200 lbs acre^-1^ (38-0-0; ~85 kg N ha^-1^) |
| Harvest | All | Sep 10, 2024 | Chopped for silage | Forage harvester with yield monitor |
| Manure application | All | Sep 24, 2024 | Broadcast (Conventional), injection (Soil Health), manure spreader (Solids) | See Table 1 |
| Cover crop planting | Soil Health, Solids | Aug 20, Aug 21, Aug 22, 2024 | Drone seeded | 100 lbs/ac |


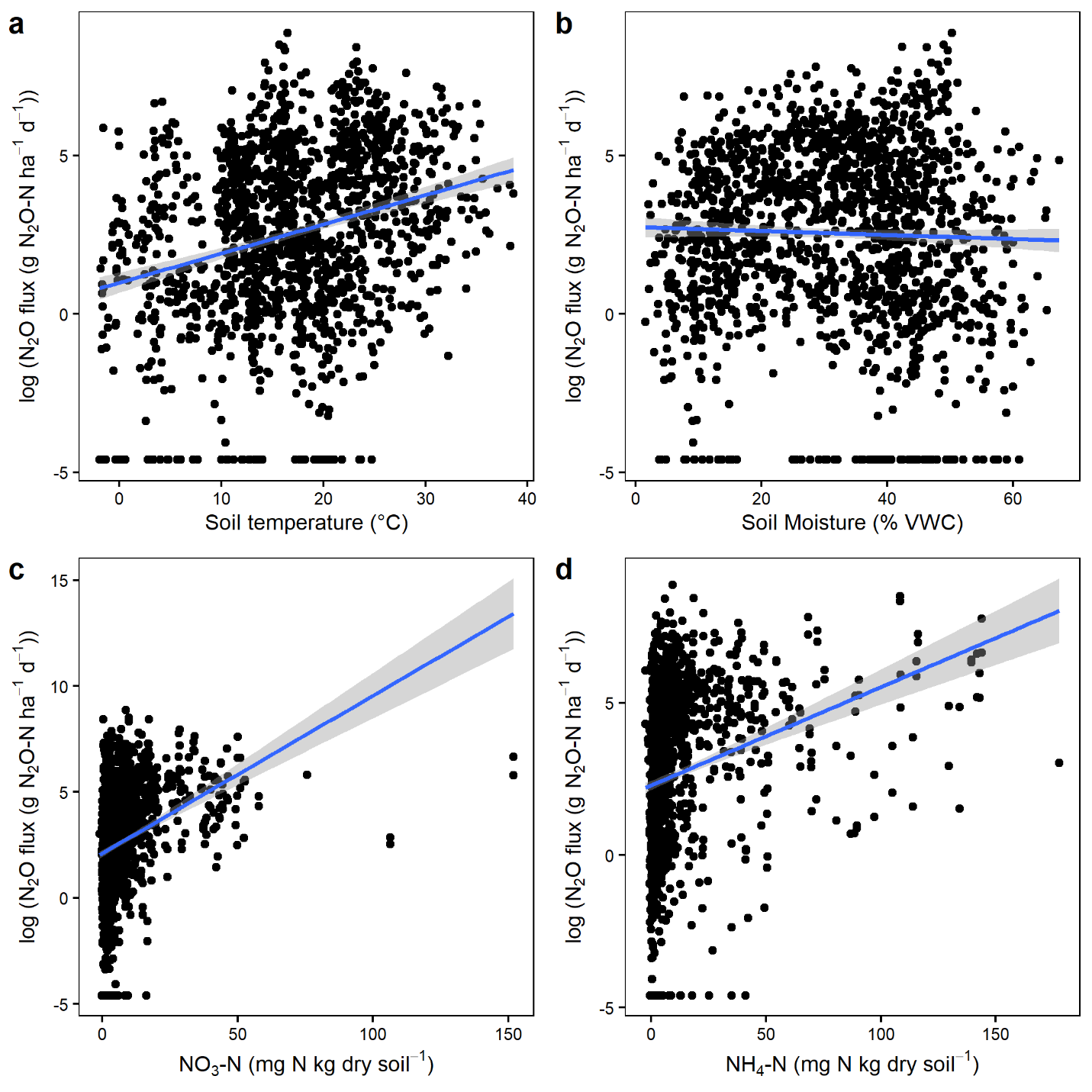


**Supplemental Figure S3.** Relationships between log transformed daily N_2_O data and covariates.

**SUPPLEMENTAL TABLE SIII** Analysis of variance (ANOVA) results for daily CO_2_ fluxes, soil moisture, soil ammonium concentrations, and soil nitrate concentrations

|  | **Daily CO_2_ Flux** | | **Soil Moisture** | | **NH_4_^+^** | | **NO_3_^-^** | |
| --- | --- | --- | --- | --- | --- | --- | --- | --- |
|  | **ANOVA** | | **ANOVA** | | **ANOVA** | | **ANOVA** | |
|  | ***F*** | ***P*** | ***F*** | ***P*** | ***F*** | ***P*** | ***F*** | ***P*** |
| Mgmt | 1.8 | 0.2 | 3.2 | 0.05 | 126.2 | < 0.0001^*^ | 56.8 | < 0.0001^*^ |
| Topo | 97.2 | < 0.0001^*^ | 114.2 | < 0.0001^*^ | 108.7 | < 0.0001^*^ | 4.0 | 0.05 |
| Mgmt x Topo | 3.7 | 0.04^*^ | 10.5 | 0.0004^*^ | 5.1 | 0.01^*^ | 2.1 | 0.2 |
| Marginal R^2^ | 0.70 |  | 0.73 |  | 0.62 |  | 0.43 |  |
| Conditional R^2^ | 0.70 |  | 0.75 |  | 0.64 |  | 0.44 |  |
| n_observations_ | 1557 |  | 1557 |  | 1471 |  | 1471 |  |

**Supplemental Figure S4.** Partial dependence plots for the top four predictor variables (> 10% relative influence) of N_2_O flux in the best iteration of the N_2_O BRT model. The y axis represents predicted N_2_O fluxes from the model. Rug plots along the x axes show distribution of observed data for each independent variable. VWC, volumetric water content.

**Supplemental Table SIV.** Analysis of variance (ANOVA) results for daily N_2_O and CH_4_ emissions using post-treatment observations only.

|  | **Daily N_2_O Flux** | | **Daily CH_4_ Flux** | |
| --- | --- | --- | --- | --- |
|  | **ANOVA** | | **ANOVA** | |
|  | ***F*** | ***P*** | ***F*** | ***P*** |
| Management | 174.4 | < 0.0001^*^ | 32.7 | < 0.0001^*^ |
| Topo | 21.9 | < 0.0001^*^ | 8.8 | 0.006^*^ |
| Management x Topo | 0.5 | 0.5 | 6.3 | 0.005^*^ |
| Marginal R^2^ | 0.70 | - | 0.25 | - |
| Conditional R^2^ | 0.70 | - | 0.25 | - |
| n_observations_ | 1240 | - | 1240 | - |

**SUPPLEMENTAL TABLE SV.** Analysis of variance (ANOVA) results for 2024 corn silage yield (Mg ha^-1^) and N_2_O intensity (kg N_2_O-N Mg^-1^ corn).

|  | **Yield** | | **N_2_O Intensity** | |
| --- | --- | --- | --- | --- |
|  | **ANOVA** | | **ANOVA** | |
|  | ***F*** | ***P*** | ***F*** | ***P*** |
| Mgmt | 2.6 | 0.1 | 9.5 | 0.003^*^ |
| Topo | 13.0 | 0.004^*^ | 16.1 | 0.002^*^ |
| Mgmt x Topo | 2.3 | 0.1 | 5.1 | 0.02^*^ |
| R^2^ | 0.57 | - | 0.73 | - |
| n_observations_ | 18 | - | 18 | - |

**SUPPLEMETNAL TABLE SVI.**  Mean (**±**SE) 2024 corn silage yield (Mg ha^-1^) and N_2_O intensity (kg N_2_O-N Mg^-1^ corn) by management treatment and topographic position.

| **Management** | **Topography** | **Yield** | **Emission Intensity** |
| --- | --- | --- | --- |
| Soil Health | Low | 20.0 ± 3.8 | 4.0 ± 0.8 |
| Soil Health | High | 33.9 ± 4.7 | 0.8 ± 0.1 |
| Conventional | Low | 14.5 ± 1.4 | 2.5 ± 0.9 |
| Conventional | High | 25.8 ± 2.4 | 1.1 ± 0.1 |
| Flocculated Solids | Low | 23.7 ± 3.0 | 0.4 ± 0.08 |
| Flocculated Solids | High | 25.2 ± 1.3 | 0.3 ± 0.05 |
